# SERT-to-DAT ratios in early Parkinson’s disease do not correlate with the development of dyskinesias

**DOI:** 10.1186/2191-219X-3-44

**Published:** 2013-06-05

**Authors:** Sven R Suwijn, Henk W Berendse, Constant VM Verschuur, Ania Winogrodzka, Rob MA de Bie, Jan Booij

**Affiliations:** 1 Department of Neurology, H2-225, Academic Medical Center, University of Amsterdam, PO Box 22660, Amsterdam 1100 DD, The Netherlands; 2 Department of Neurology, VU University Medical Center, PO Box 7057, Amsterdam, 1007 MB, The Netherlands; 3 Department of Neurology, Medical Center Alkmaar, PO Box 501, Alkmaar 1800 AM, The Netherlands; 4 Department of Nuclear Medicine, Academic Medical Center, PO Box 22660, Amsterdam 1100 DD, The Netherlands

**Keywords:** Parkinson’s disease, Dopamine transporter, Serotonin transporter, [^123^I]β-CIT SPECT, Dyskinesias, Age of onset

## Abstract

**Background:**

Although the treatment of Parkinson’s disease (PD) is very effective, in the course of the disease, 40% to 60% of patients develop dyskinesias. The pathophysiology of dyskinesias is still unclear. Results of preclinical research suggest that uptake and uncontrolled release of dopamine by serotonergic neurons is an important factor. Based on this model, we hypothesized that dyskinesias will develop predominantly in PD patients with a relatively preserved serotonergic system.

**Methods:**

Between 1995 and 1998, 50 patients with early-stage untreated PD, diagnosed according to clinical criteria, and reduced striatal [^123^I]β-carboxymethyoxy-3-beta*-*(4-iodophenyl) tropane (CIT) single-photon emission computed tomography (SPECT) binding were recruited. To test our hypothesis, we retrospectively assessed baseline [^123^I]β-CIT SPECT scans for striatal dopamine transporter (DAT) and midbrain serotonin transporter (SERT) availability as well as the SERT-to-DAT ratios. We compared these data between patients that developed dyskinesias and patients that did not develop dyskinesias during a mean follow-up of 14.2 years.

**Results:**

Approximately half of the PD patients developed dyskinesias. No differences in baseline [^123^I]β-CIT DAT availability, SERT availability, or SERT-to-DAT ratios were found between the dyskinetic and non-dyskinetic group. The development of dyskinesias was most strongly associated with the age of onset (*P* = 0.002).

**Conclusions:**

SERT-to-DAT ratios in early-stage untreated PD do not correlate with the future development of dyskinesias. However, our study does not exclude the possibility that SERT-to-DAT ratios increase with disease progression in patients that develop dyskinesias because of a slower rate of degeneration of the serotonergic system.

## Background

Parkinson’s disease (PD) is the second most common form of neurodegenerative diseases [1]. A key neuropathological characteristic of PD is a severe loss of dopamine-producing neurons in the brainstem, which induces several core motor features such as bradykinesia and rigidity. The development of levodopa is a milestone in the treatment of PD, since it is inexpensive and very efficacious [2]. Levodopa is converted to dopamine which replenishes the stores of endogenous dopamine and induces a fast and significant improvement in motor function [3]. Due to disease progression, patients require higher daily dosages of levodopa to produce a stable clinical effect. Frequently, disabling side-effects in particular dyskinesias occur [4]. After 5 years of levodopa treatment, approximately 30% to 40% of patients suffer from dyskinesias, increasing to 40% to 60% after 10 years of treatment [5,6]. Nevertheless, the pathophysiology of dyskinesias is still unknown. There is a clear need for a better understanding of the pathophysiology, which may yield novel targets to develop improved treatment strategies.

In the brain, the pathway that projects from the substantia nigra to the striatum is the most prominent dopaminergic pathway. The central serotonergic system originates in the raphe nuclei. Within this nuclei complex, the dorsal raphe nucleus projects predominantly to cortical areas and the striatum [7]. PD is not only characterized by dopaminergic degeneration but also by serotonergic degeneration [8,9]. Preclinical research has led to the development of a model that has the potential to explain the development of dyskinesias [10]. This model postulates that an imbalance between dopamine and serotonin plays a crucial role in the development of dyskinesias. More specifically, the model presumes that in early stage PD, sufficient dopaminergic neurons exist to regulate and release dopamine adequately. As the disease progresses, the number of dopaminergic neurons declines. The loss of serotonin neurons, however, is relatively mild compared to the loss of dopamine neurons [8,11]. Terminal of serotonergic neurons in the striatum can also take up, store, and release dopamine, yet these neurons lack auto-regulatory feedback mechanisms of dopaminergic neurons to release dopamine adequately (serotonergic neurons lack D_2_ auto-receptors and dopamine transporters) [12,13]. As a result of the lack of these mechanisms, dopamine release from serotonin nerve terminals in PD may be poorly regulated, resulting in uncontrolled excessive swings in dopamine release (called release of ‘false transmitter’).

In line with the model described above, studies in dopamine-depleted rats and 1-methyl-4-phenyl-1,2,3,6-tetrahydropyridine-treated non-human primates have shown that the removal of serotonin neurons and/or reduction of serotonin activity by serotonin agonists resulted in a significant decrease of dyskinesias [10,14]. More specifically, the blockade of serotonin neuron activity by the combination of 5-HT1A agonists prevented the unregulated dopamine release by central serotonergic neurons and consequently prevented the development of dyskinesias in dopamine-depleted rats [15].

Furthermore, an increase in the incidence of dyskinesias has been observed in dopamine-depleted rats that received a transplant containing relatively many serotonin and few dopamine cells, whereas the dyskinesias decreased when rats received a transplant consisting predominantly of dopaminergic neurons [16]. An increase of dyskinesias was also observed in two PD patients who received a graft with a high striatal serotonin/dopamine transporter ratio. Moreover, administration of a serotonin 1A receptor agonist (buspirone) significantly reduced the severity of dyskinesias in both patients [17].

### Hypothesis

All in all, particularly the results of the preclinical studies suggest that dyskinesias may predominantly develop in PD patients with a relative spared serotonergic system. This study aims to determine whether an imbalance between the loss of dopaminergic and serotonergic neurons precedes the development of dyskinesias in PD. Therefore, we retrospectively assessed striatal dopamine transporter (DAT) and midbrain serotonin transporter (SERT) availability as well as the SERT-to-DAT ratios, as measured with [^123^I]β-carboxymethyoxy-3-beta*-*(4-iodophenyl) tropane (CIT) (a radiotracer that binds to both the DAT and SERT [18]) single-photon emission computed tomography (SPECT) in drug-naïve early stage PD patients. We compared these data between patients who had developed dyskinesias and patients who had not developed dyskinesias during a minimum of 5-year follow-up. We expected that PD patients who had developed dyskinesias would have baseline [^123^I]β-CIT SPECT scans with higher SERT-to-DAT ratios.

## Methods

### Subjects

A total of 77 patients with early-stage untreated PD, diagnosed according to the United Kingdom Parkinson’s Disease Society Brain Bank clinical diagnostic criteria [19] and reduced striatal [^123^I]β-CIT SPECT binding at the time of the initial clinical diagnosis were recruited from the outpatient clinic for movement disorders at the VU University Medical Center (VUMC) between 1995 and 1998. In all patients, [^123^I]β-CIT SPECT imaging was performed before the initiation of dopaminomimetic therapy. None of the subjects was using a compound that potentially interferes with [^123^I]β-CIT SPECT binding. Furthermore, all medical records were reviewed to ensure none of the patients was taking serotonergic medication at the time of SPECT imaging. At the time of diagnosis, the Beck Depression Inventory scale was used to ensure none of the patients had signs of a major depressive disorder. PD patients with dementia at baseline were not included; a mini-mental state examination score below 26 was used as an exclusion criterion. Disease duration was based on the dates of the clinical diagnosis by a neurologist and the last contact during follow-up. The onset of dyskinesias was defined as the first time the neurologist reported the presence of dyskinesias. All subjects gave written informed consent to the research protocol, which was approved by the local medical ethical committee of the VUMC. The ethical review criteria conformed to the declaration of Helsinki.

**Figure 1:**
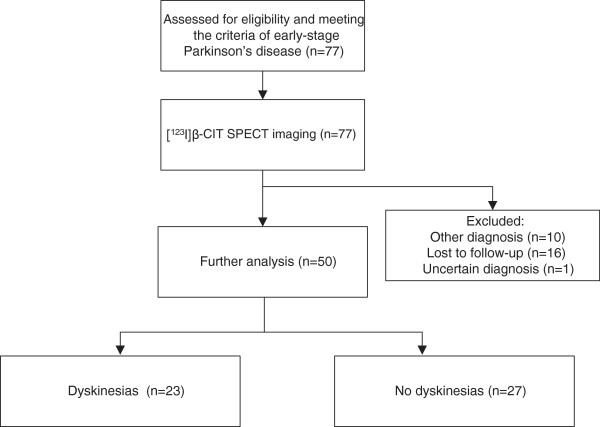
Flow chart of patients’ disposition.

In ten patients out of the total group of 77 patients, the clinical diagnosis was changed during follow-up, as previously reported [20], and these were excluded from the present study (Figure 1). All patients were followed for at least 5 years. Sixteen patients were lost to follow-up, and 1 had an uncertain diagnosis, leaving a group of 50 patients for further analysis.

### SPECT imaging

SPECT imaging was performed using a brain-dedicated system, the SME 810X system (Strichmann Medical Equipment Inc., Medfield, MA, USA). This system consists of 12 individual crystals each equipped with a focusing collimator. The spatial resolution of this camera system is approximately 6.5 mm full-width at half-maximum, throughout the 20-cm field of view. In order to block the thyroid uptake of free radioactive iodine, the subjects received potassium iodide orally. [^123^I]β-CIT (specific activity >185 MBq/nmol, radiochemical purity >99%) was injected intravenously at an approximate dose of 110 MBq. [^123^I]-labeling and acquisition were performed as described previously [21]. Image acquisition was performed 24 h after injection. Images were corrected for attenuation and reconstructed in 3D [22,23].

### Analysis of images

For the analysis of striatal and midbrain [^123^I]β-CIT binding, two consecutive transverse slices representing the most intense striatal and midbrain binding, were analyzed. A standard anatomical region-of-interest (ROI) template (constructed according to a stereotactic atlas) and including regions for the caudate nucleus, putamen, whole striatum, midbrain, and occipital cortex (representing non-specific binding) was placed bilaterally on the images, as previously reported [24]. Estimates of specific striatal binding were made by subtracting occipital counts from striatal counts. Specific [^123^I]β-CIT binding ratios in striatum, caudate nucleus, putamen, and midbrain were calculated using the formula: (mean binding in ROI-mean occipital binding) / mean occipital binding. This formula is referred as binding potential non-displaceable (BP_ND_) [25].

Furthermore, to assess if a higher SERT-to-DAT ratio precedes the development of dyskinesias, we calculated the ratios of midbrain BP_ND_ versus BP_ND_ assessed in the caudate nucleus, putamen, and whole striatum. We also calculated these ratios in the most affected side [17].

### Statistical analysis

The Kolmogorov-Smirnov test was applied to screen for normality. Group differences in the distribution of gender, side-of-onset, and modified Hoehn and Yahr scores were analyzed by means of the chi-square test. Analysis with regard to group difference in time from diagnosis until start of levodopa was analyzed using the Mann–Whitney *U* test. Possible differences in age, age of onset, disease duration, time between start of levodopa and the development of dyskinesias, years of levodopa use, and the unified Parkinson’s disease rating scale (UPDRS) motor scores were analyzed using the independent *t* test. In addition, the UPDRS motor score was divided into two subscores that represented predominantly dopaminergic (subscore A) and non-dopaminergic (subscore B) deficiency [26]. We compared the [^123^I]β-CIT BP_ND_ values and SERT-to-DAT ratios between the group with dyskinesias (PD_DYS_) and without dyskinesias (PD_NDYS_) using one-way analysis of variance. After transformation by means of a natural logarithm, all met with the assumption of normality. We intended to perform logistic regression to assess the impact of the different variables on the chance of developing dyskinesias. Considering that the smallest group consisted of 23 patients, only 2 variables could be used for logistic regression. Therefore, we used the independent *t* test to analyze the variables independently. All analyzes were performed at a significance level of 0.05 (two-tailed). Analysis was done using the SPSS 20.0 software package (IBM SPSS Inc., Chicago, IL, USA).

## Results

### Patients

The demographic and clinical characteristics are listed in Table 1. Twenty-three patients (46%) developed dyskinesias, and 27 (54%) did not. Twelve patients died during follow-up (ten in PD_NDYS_ and two in the PD_DYS_ group). The mean follow-up of the deceased patients was 11.8 years age of onset (*P* = 0.002), disease duration (*P* = 0.003), UPDRS motor score at the moment of imaging (*P* = 0.008), the total follow-up period (*P* = 0.004), and years of levodopa use (*P* < 0.001), were significantly different between the PD_DYS_ and PD_NDYS_ groups. There were no differences regarding the other clinical characteristics.

**Table 1:** Demographic and clinical characteristics

	**PD**_**NDYS **_**(*n *= 27)**	**PD**_**DYS **_**(*n *= 23)**	***P *value**
Age of onset PD (mean ± SD) (year)	56.8 ± 6.8	49.7 ± 10.9	0.002
Male sex number (percent)	15 (56)	16 (70)	0.39
Total follow-up (mean ± SD) (months)	155 ± 49	190 ± 29	0.004
Most affected side (striatal) (right/left)	11/16	11/12	0.43
Disease duration (mean ± SD) (year)	12.8 ± 4.1	15.8 ± 2.4	0.003
Disease duration until levodopa use (median, ICR) (year)	3.0, 4.75	2.4, 2.6	0.57
Disease duration until onset dyskinesias (mean ± SD) (year)	NA	7.8 ± 3.9	NA
Levodopa use until onset dyskinesias (mean ± SD) (year)	NA	5.1 ± 3.0	NA
Levodopa use (mean ± SD) (year)	9.5 ± 3.4	13.2 ± 2.0	<0.001
UPDRS motor score at moment of imaging	16.9 ± 6.4	21.4 ± 5.1	0.008
(total range, 0 to 108) (mean ± SD)			
Subscore A (0 to 88)	10.8 ± 3.4	14.2 ± 3.4	0.001
Subscore B (0 to 20)	6.1 ± 3.9	7.2 ± 2.8	0.25
Modified HY score at moment of imaging (1/1.5/2/2.5)	7/3/8/9	6/1/9/7	0.79

*PD* Parkinson’s disease, *PD*_*NDYS*_ PD patients subgroup that did not develop dyskinesias, *PD*_*DYS*_ PD patients subgroup that did develop dyskinesias, *SD* standard deviation, *ICR* interquartile range, *NA* data not available, *UPDRS* unified Parkinson’s disease rating scale, UPDRS subscore A, consists of dopaminergic symptoms, subscore B, consists of non-dopaminergic symptoms, *HY* Hoehn and Yahr.

### Analysis of [^123^I]β-CIT SPECT binding

**Figure 2:**
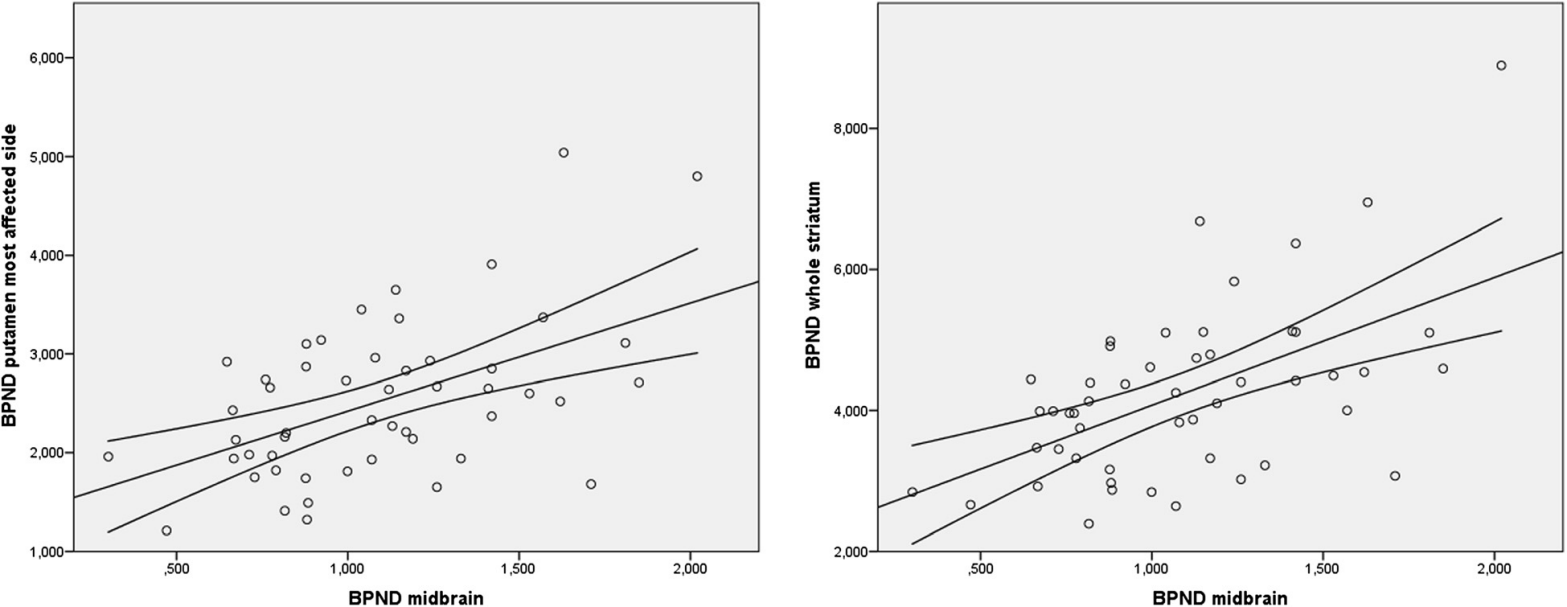
**Correlation between serotonin and dopamine transporter binding.** Serotonin transporter binding (BP_ND_ midbrain) is significantly correlated with dopamine transporter binding in the most affected putamen (BP_ND_ putamen most affected side, left panel; *r* = 0.496, *P* ≤ 0.001) and striatum (BP_ND_ striatum, right panel; *r* = 0.532, *P* ≤ 0.001).

The mean [^123^I]β-CIT BP_ND_ in the putamen was reduced compared to the caudate nucleus, the typical pattern of degeneration in PD. In the whole group, BP_ND_ in the midbrain correlated significantly (Pearson’s correlation) with BP_ND_ in the most affected putamen (*r* = 0.496, *P* < 0.001) and whole striatum (*r* = 0.532, *P* < 0.001; Figure 2).

No differences in baseline [^123^I]β-CIT BP_ND_ were found between the PD_DYS_ and PD_NDYS_ group (Table 2). This was true for BP_ND_ in the caudate nucleus, putamen, whole striatum (mean for both sides, as well as for the most affected side), and in the midbrain. Also, no significant differences between PD_NDYS_ and PD_DYS_ in SERT- to-DAT ratios were observed (Table 2). Also, BP_ND_ in the caudate nucleus, putamen, striatum, and midbrain did not predict the development of dyskinesias.

**Table 2:** **Mean specific to non-specific [**^**123**^**I]β-CIT binding ratios (BP**_**ND**_**) (mean** ± **SD) and SERT-to-DAT ratios**

**Region of interest**	**PD**_**NDYS **_**(*n *= 27)**	**PD**_**DYS **_**(*n *= 23)**	***P *value**
Striatum, whole	4.02 ± 0.30	4.16 ± 0.24	0.66
Striatum, most affected side	3.43 ± 0.31	3.59 ± 0.27	0.57
Caudate nucleus, whole	6.02 ± 0.30	6.33 ± 0.25	0.52
Caudate nucleus, most affected side	5.34 ± 0.34	5.73 ± 0.28	0.43
Putamen, whole	3.03 ± 0.32	3.00 ± 0.26	0.93
Putamen, most affected side	2.39 ± 0.33	2.43 ± 0.28	0.82
Midbrain	1.05 ± 0.40	1.14 ± 0.35	0.41
Midbrain/Putamen, whole	0.33 ± 0.32	0.35 ± 0.36	0.48
Midbrain/Putamen most affected side	0.42 ± 0.32	0.44 ± 0.38	0.67
Midbrain/Caudate, whole	0.17 ± 0.32	0.17 ± 0.32	0.88
Midbrain/Caudate, most affected side	0.19 ± 0.34	0.19 ± 0.37	0.97
Midbrain/Striatum, whole	0.25 ± 0.31	0.26 ± 0.34	0.75
Midbrain/Striatum, most affected side	0.29 ± 0.31	0.30 ± 0.38	0.87

*SERT* serotonin transporter, *DAT* dopamine transporter, *SD* standard deviation, *PD* Parkinson’s disease, *PD*_*NDYS*_ PD patients subgroup that did not develop dyskinesias, *PD*_*DYS*_ PD patients subgroup that developed dyskinesias.

## Discussion

This is the first study to assess SERT-to-DAT ratios in a relatively large cohort of drug-naive patients with early stage PD and a mean follow-up of 14 years. During a mean follow-up of 14 years, 46% of our patients developed dyskinesias, which is in accordance with the existing literature [5,6]. Patients in the PD_DYS_ group had longer mean disease duration and consequently had longer follow-up than patients in the PD_NDYS_ group. This could be explained by the uneven distribution of deceased patients (ten in the PD_NDYS_ and two in PD_DYS_ group) and the lower age of onset in the PD_DYS_ group. Also, at the moment of imaging, patients in the PD_DYS_ group had higher UPDRS motor scores than patients in the PD_NDYS_ group. The difference in UPDRS scores was caused by a difference in dopaminergic rather than non-dopaminergic symptoms (Table 2). Interestingly, this subgroup of patients was younger at disease onset, so one can speculate that younger patients often still work and have busy lives and therefore may not notice the subtle motor signs of PD.

Our study confirms that the age of onset of disease is an independent risk factor for developing dyskinesias. The two groups had a mean age of PD onset of 57 (PD_NDYS_) and 50 years (PD_DYS_), respectively. This observation is in line with previous reports, which showed that the 5-year incidence of dyskinesias in newly diagnosed PD patients is age of onset-dependent: 50% between the ages of 40 and 59 years, 26% between the ages of 60 and 69 years, and 16% at 70 years and older [6]. Furthermore, our patients who developed dyskinesias started on average of 0.6 years earlier with levodopa compared to the PD_NDYS_ group (3.0 and 2.4 years, respectively) due to the longer disease duration, hence the difference in duration of levodopa use.

The hypothesis is that serotonergic neurons lack the feedback mechanisms of dopaminergic neurons to release dopamine adequately [12,13]. As a consequence, dopamine release from serotonin nerve terminals will be poorly regulated, resulting in uncontrolled, excessive swings in dopamine release. In contrast to this hypothesis, in the present study, the dyskinesias were not preceded by a higher SERT-to-DAT ratio in patients with dyskinesias compared to non-dyskinetic patients. Our findings, however, do not necessarily reject the hypothesis that dyskinesias are associated with a relatively preserved serotonergic system. More specifically, since we performed a [^123^I]β-CIT scan at baseline, we cannot exclude the possibility that a change in SERT-to-DAT ratio occurs later in the course of the disease as a result of a slower progression of the degeneration of the serotonergic system compared to that of the dopaminergic system, particularly in patients that go on to develop dyskinesias. In this regard, it is of interest that a recent study using positron emission tomography (PET) and the selective SERT tracer [^11^C]labeled 3-amino-4-(2-dimethylaminomethyl-phenylsulfanyl)benzonitrile (DASB) demonstrated a non-linear loss of presynaptic serotonergic neurons across the clinical course of PD. In the early stages of PD (disease duration shorter than 5 years), [^11^C]DASB uptake was reduced only in the caudate nucleus, hypothalamus, thalamus, and anterior cingulate cortex. In established PD (disease duration 5 to 10 years), the uptake was additionally reduced in the putamen, insular cortex, prefrontal cortex, and posterior cingulate cortex [27]. Furthermore, in other studies, loss of dopaminergic input to the putamen was severely reduced in early PD stages, while the loss of [^11^C]DASB uptake in the putamen occurred later [28,29]. These data are in support of the assumption that serotonergic degeneration occurs at a different/slower rate in patients who develop dyskinesias compared to patients that do not.

DAT binding in PD is commonly asymmetric, and the loss of binding is generally more profound in the putamen than in the caudate nucleus, which is also the case in this study, thus confirming the findings of earlier DAT SPECT studies in PD [20,23]. We hypothesized that if dopamine would be taken up predominantly by serotonergic neurons, then this phenomenon would first occur in the most affected striatal area. In other words, if high SERT-to-DAT ratios precede the development of dyskinesias, one may postulate that SERT-to-DAT ratios will be highest in the most affected putamen. However, also in this part of the striatum, SERT-to-DAT ratio was almost similar (Table 2) between the two groups.

Although it is not possible to measure SERT availability in the striatum using [^123^I]β-CIT, midbrain [^123^I]β-CIT binding is mainly associated with the binding to SERT, while binding in the striatum is mainly associated with binding to DAT [24]. Therefore, DAT and SERT availability can be measured accurately with [^123^I]β-CIT SPECT in different areas of the brain. However, in the present study, SERT binding in the midbrain correlated significantly with DAT binding in the most affected putamen and whole striatum (Figure 2). This finding is in line with a previous PET study which reported a positive correlation between DAT and SERT binding in the striatum of PD patients [30]. Although such a positive correlation could not be replicated in a later smaller study [31], this might indicate that SERT expression in the midbrain and striatum are associated. Unfortunately, in the period in which the participants were recruited and imaged, selective SERT tracers for SPECT, like [^123^I]ADAM, were not available to assess SERT binding in the striatum [32]. On the other hand, both [^123^I]FP-CIT and [^123^I]β-CIT have been used to image SERT and DAT in different brain areas [23,33], though the DAT/SERT selectivity is somewhat lower for β-CIT (1.7:1 and 2.8:1, respectively), which favors the use of [^123^I]β-CIT over [^123^I]FP-CIT to assess extrastriatal SERT binding [34].

Our study has both strengths and limitations. Patients were screened to exclude the presence of major depressive disorders that might have had a possible confounding effect. Furthermore, we reviewed the medical charts, with the current knowledge, to determine whether any of the patients was using a compound that may have interfered with [^123^I]β-CIT binding at the time of diagnosis. Moreover, all scans were acquired before any dopaminergic medication was initiated to reduce possible confounding effects.

Due to the small group size (*n* = 23) an accurate analysis using logistic regression to assess the impact of other variables (limited to two covariates) was not possible. However, the BP_ND_ in striatal and midbrain areas were not even close to a significant difference between groups (Table 2). Therefore, it is unlikely that a larger prospective study would prove that the SERT-to-DAT ratio in early stage drug-naïve PD patients correlates with the development of dyskinesias. Another potential limitation of this study is the retrospective collection of dyskinesia data and the accompanying disadvantages thereof. However, most of the patients were closely monitored.

## Conclusions

In conclusion, we found that the development of dyskinesias is not associated with baseline striatal DAT, midbrain SERT availability, or higher SERT-to-DAT ratios. In addition, we confirmed that development of dyskinesias is age-of-onset-dependent. The first finding does not support preclinical data suggesting an influence of relative sparing of the serotonergic system. However, we cannot exclude that the progression of degeneration of the serotonergic system is slower in PD_DYS_ compared to PD_NDYS_, ultimately resulting in higher SERT-to-DAT ratios at the onset of dyskinesias. Prospective imaging studies, using selective radiotracers for the SERT and DAT, are needed to shed more light on the presumed role of a relatively preserved serotonergic system in the induction of dyskinesias.

## Competing interests

The authors declare that they have no competing interests.

## Authors’ contributions

SRS participated in the research project organization and execution and in the design and execution of the statistical analysis, data collection, and writing of the first draft of the manuscript. HWB and AW participated in the review and critique on the manuscript and data collection. CVMV participated in the review and critique on the manuscript. RMADB conceived of the research project. JB participated in the research project conception, organization, and execution, data collection, and review and critique on the statistical analysis and manuscript. All authors read and approved the final manuscript.
